# Variation in cloacal microbiota of Canada goose (*Branta canadensis*) across rural and urban areas in Illinois, USA

**DOI:** 10.3389/frmbi.2026.1820309

**Published:** 2026-06-17

**Authors:** Daniel B. Raudabaugh, Sara Villazan Perez-Girones, Nelda A. Rivera, Tooba Latif, Evan W. London, Nicole F. Pietrunti, Willian M. Brown, Nohra E. Mateus-Pinilla, Auriel M. V Fournier

**Affiliations:** 1 Department of Botany and Plant Pathology, Purdue University, University of Illinois Urbana-Champaign, Urbana, IL, United States; 2 Illinois Natural History Survey, Prairie Research Institute, University of Illinois Urbana- Champaign, Urbana, IL, United States; 3 Department of Biology, University of Central Oklahoma, Edmond, OK, United States; 4 Department of Animal Sciences, University of Illinois Urbana-Champaign, Urbana, IL, United States; 5 Forbes Biological Station–Bellrose Waterfowl Research Center, Illinois Natural History Survey, Prairie Research Institute, University of Illinois-Champaign, Urbana, IL, United States; 6 Department of Natural Resources and Environmental Sciences, University of Illinois Urbana-Champaign, Urbana, IL, United States; 7 Department of Pathobiology, University of Illinois Urbana-Champaign, Urbana, IL, United States

**Keywords:** bacterial disease, microbiomes, urban, waterfowl, wildlife

## Abstract

**Introduction:**

The cloacal microbiota of birds is shaped by host factors, diet, environmental exposure, and increasing overlap between wild bird habitats and human development may influence these communities. However, the effects of urbanization on herbivorous waterfowl in Illinois remain poorly understood.

**Methods:**

In this study, we characterized the cloacal microbiota of 106 Canada goose (*Branta canadensis*) sampled from rural and urban areas in Illinois using 16S rRNA gene V4 amplicon sequencing, and evaluated associations between host age, host sex, and human population density and microbial community structure.

**Results:**

The cloacal microbiota included 29 phyla, 56 classes, and at least 131 orders, and was dominated by Bacillota, Actinomycetota, Pseudomonadota, and Bacteroidota. Common gutassociated taxa included *Clostridium, Ruminococcus*, and *Eubacterium*, whereas plant- and soil-associated bacteria, including nitrogen-fixing members of the Rhizobiaceae, likely reflect dietary and environmental acquisition during foraging. Alpha diversity metrics did not differ significantly across host age or sex, although ASV richness was significantly higher in rural compared to urban samples. In contrast, beta-diversity analyses indicated that host age was the strongest factor associated with differences in microbial community composition, with additional but weaker effects of human population density, while host sex had comparatively little influence.

**Discussion:**

Overall, these results suggest that ecological context, including habitat type and environmental exposure, were associated with variation in the cloacal microbiota of Canada goose, although additional unmeasured environmental and spatial factors may also contribute to observed patterns. This study provides a baseline characterization of microbiota variation across age classes and habitats in Illinois Canada goose and highlights the importance of considering ecological context when interpreting wildlife-associated microbial communities.

## 1 Introduction

The microbiota of animals, the complex community of viruses, bacteria, fungi, archaea, and protozoa that live on and within them, participates in and is often considered essential for normal host physiological function and disease resistance (Marchesi and Ravel, 2015; Safika et al., 2024). Moreover, behavior change and the local environment may contribute to shifts in the microbiota. *Branta canadensis* (Canada goose) are typically migratory North American birds; however, some populations have become resident and now breed within Illinois (Sertle and Eichholz, 2006). These resident populations persist across both urban and rural environments, where they exploit a wide range of anthropogenic and natural habitats. Because of their ecological flexibility and frequent use of human-altered landscapes, Canada goose may serve as useful sentinels of environmental microbial conditions, including potential exposure to opportunistic or pathogenic bacteria in shared environments such as parks, waterways, and waste-associated sites.

Despite this potential, research on the gut microbiota of wild birds remains comparatively limited compared with that of humans and agriculturally important species (Suchodolski, 2018; Waite and Taylor, 2015). Most avian microbiome studies have focused primarily on bacterial communities, with relatively few addressing broader ecological or geographic drivers. In Canada goose specifically, previous work has examined microbial communities across gastrointestinal regions and fecal samples from multiple U.S. states, including Connecticut, Washington, New York, New Jersey, Rhode Island, Kansas, Utah, Nevada, and the District of Columbia (Drovetski et al., 2018; Gil and Hird, 2022, Gil et al., 2025). These studies demonstrate that geography and habitat can influence microbial composition. However, they also highlight the need for additional population-level sampling across underrepresented regions, including the Midwest and urban centers such as Illinois.

Ecological theory and prior microbiome research suggest that microbial community structure is shaped by the interaction of: 1) environmental context, 2) diet-mediated microbial acquisition, and 3) host demographic traits. For example, in humans, microbial communities vary with diet and undergo predictable shifts across the lifespan, with early-life communities characterized by lower diversity and age-associated restructuring over time (David et al., 2014; O’Toole and Jeffery, 2015; Yatsunenko et al., 2012). Geographic variation also contributes substantially to microbial differences, driven by differences in climate, diet, and environmental exposure, with reduced diversity often observed in more industrialized or urbanized settings (Schnorr et al., 2014; Yatsunenko et al., 2012). Similar patterns have been observed in animals, where habitat type and foraging ecology strongly influence gut microbial composition (Amato et al., 2013).

Environmental sequencing is a powerful tool that scientists use to study microbiota by extracting DNA from a sample, amplifying a target gene region, and sequencing the resulting amplicons on a high-throughput platform. The Illumina platform is the most widely used for this purpose, providing accurate, deep coverage that captures both abundant and rare community members (Caporaso et al., 2012). For bacterial microbiota studies, researchers typically target hypervariable regions of the 16S rRNA gene (most commonly V3–V4, V4, or V1–V3) which enables reliable taxonomic assignment and broad comparability across studies (Johnson et al., 2019; Klindworth et al., 2013). The V4 region of the 16S rRNA gene is recommended by the Earth Microbiome Project (https://earthmicrobiome.ucsd.edu/protocols-and-standards/16s/accessed 12/3/2025). It provides a good balance of taxonomic resolution across diverse bacterial phyla, with fewer biases than other 16S rRNA gene hypervariable regions (Caporaso et al., 2012; Klindworth et al., 2013; Walters et al., 2016).

Building on this framework, we hypothesize that: (1) microbial community composition will differ between urban and rural habitats due to environmental filtering and differences in resource availability; (2) host age will be associated with differences in microbial community composition, reflecting variation in microbiota assembly across life stages; and (3) host sex will contribute minimally to microbiota variation relative to environmental and age-related factors. Accordingly, microbiota variation in Canada goose is expected to reflect a combination of habitat-driven exposure and host life-history stage, particularly in terms of community composition, with potential implications for their role as indicators of microbial conditions in human-influenced environments.

## 2 Methods

### 2.1 Sampling and sample selection

**Figure 1:**
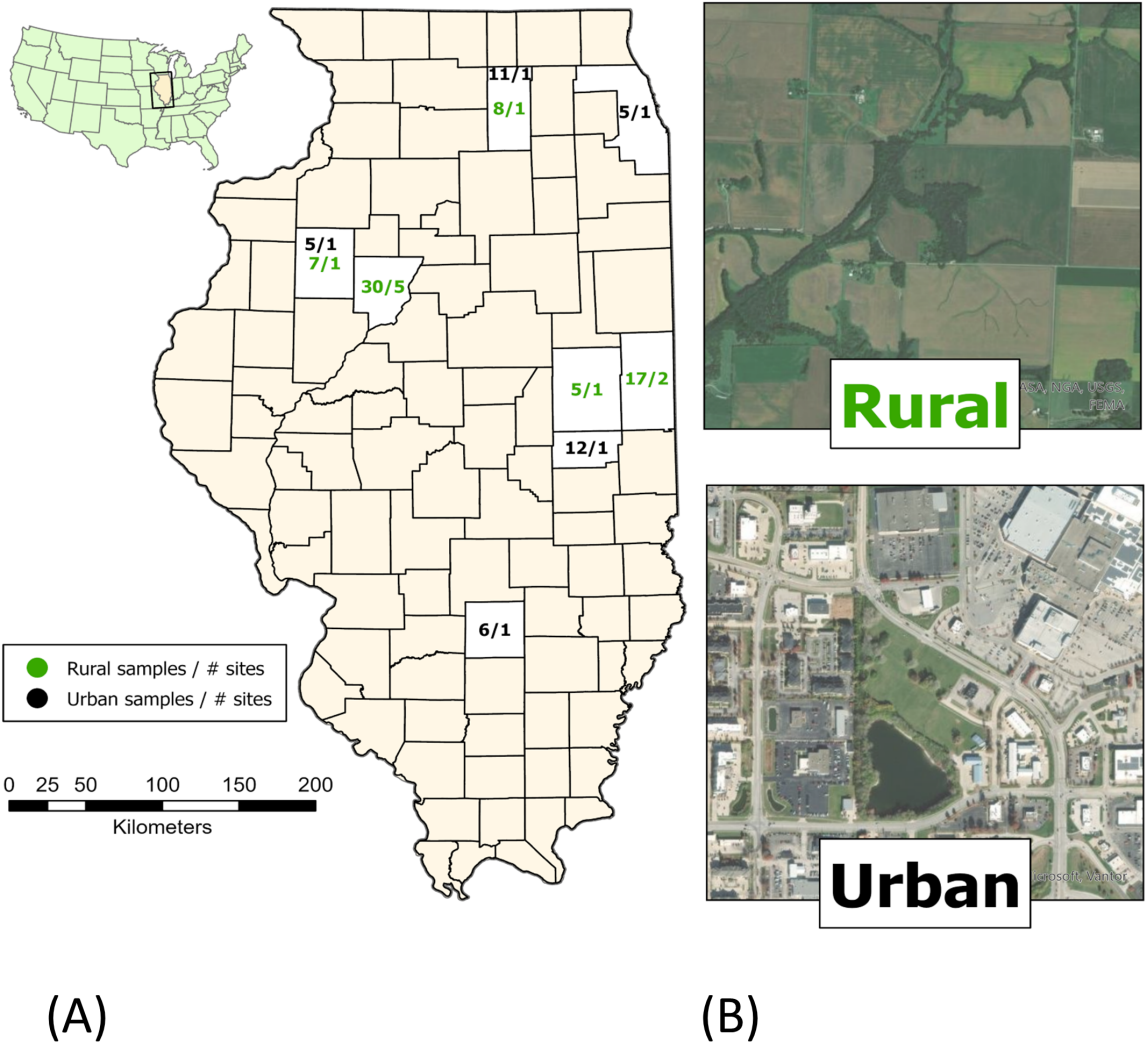
Wild geese sampling sites in Illinois, USA. Cloacal swabs were collected in 8 counties throughout the state in **(A)** Rural (sites with <50 people/sq km) and **(B)** urban (sites with >50 people/sq km). Source: Generated using ESRI ArcGIS Pro 3.5 "World Imagery" [basemap]. Scale not given. "ArcGIS Online World Imagery", November 2025, www.arcgis.com (Accessed November 4, 2025).

Capture, handling and sampling of wild geese was performed under methods approved by the University of Illinois Urbana-Champaign Institutional Animal Care and Use Committee (IACUC #22068). Cloacal swab samples were obtained from sites in eight Illinois counties in June, July, and August 2022 in coordination with the Illinois Department of Natural Resources’ Canada Goose Banding Program and the United States Department of Agriculture Animal and Plant Health Inspection Service during the brood-rearing period (**Figure 1**). The Illinois DNR bands geese annually in late June while the birds are molting their flight feathers and are unable to fly. Banding efforts were distributed across the state to band both adult (> 1 year old) and juvenile geese (< 1 year old). To capture each flock, a series of fence panels and other barriers were used to corral the birds. Individuals were then removed from the enclosed area, banded, aged based on molt examination, and sexed via visual inspection of the cloaca for the presence of a penis (Hanson, 1962).

Although a formal health assessment was not conducted for each individual, birds exhibiting obvious signs of poor condition or illness would likely have been detected and flagged by experienced personnel from the Illinois Department of Natural Resources during handling and processing.

Sites where geese were trapped were classified as Urban or Rural using a schema analogous to that employed by the United States Census Bureau, incorporating both human population density and impervious surface coverage. For each site, human population density was calculated within a 3 km radius, and impervious surface percentage was quantified within a 500 m buffer. Cutoff values for both variables were selected at natural breaks in the data, resulting in clear separation between categories. Urban sites had a mean population density of 480 people/km² (range: 55–1608) and a mean impervious surface coverage of 50% (range: 9–84). In contrast, Rural sites exhibited a mean population density of 10 people/km² (range: 3–14) and a mean impervious surface coverage of 3% (range: 1–6). Notably, the lowest population density classified as Urban (55 people/km²) corresponded to O’Hare Airport in Chicago, highlighting the importance of incorporating impervious surface metrics to accurately capture anthropogenic influence. Classifications were further validated through visual inspection of aerial imagery, as well as consideration of hydrography and watershed delineation for each site. These evaluations supported the robustness and ecological relevance of the Urban–Rural designations.

Each Canada goose was placed in a restraining hold, and two samples were collected from the cloaca using sterile swabs. The swabs were placed into the same labeled tube, kept on ice, transported back to the laboratory, and stored at –80 °C until DNA extraction. Individual geese were selected at random, with sampling balanced to obtain a similar number of adults and juveniles. In total, 106 samples were utilized for microbiota analysis in addition to two laboratory negative controls.

### 2.2 DNA extraction, PCR, and sequencing

DNA was extracted using the QIAamp PowerFecal Pro DNA Kit (Qiagen, Hilden, Germany) according to the manufacturer’s instructions. The tips of the swabs containing each sample were placed into individual wells, and nuclease-free water (50 µL) was used as a negative control in each DNA extraction bead plate (2 in total). The bead plates were shaken for 5 min at 25 Hz using TissueLyser II (Qiagen, Hilden, Germany). After initial shaking, plates were rotated and shaken for an additional 5 min. at 25 Hz to ensure uniform disruption of samples. Extracted DNA was quantified using the Invitrogen Qubit™ 4 dsDNA High Sensitivity (HS) fluorometry assay kit (Thermo Fisher Scientific), and DNA quality was assessed on a 1% TAE agarose gel stained with ethidium bromide and visualized under ultraviolet light.

Library preparation and sequencing of the cloacal swab samples and negative controls were carried out at the Roy J. Carver Biotechnology Center at the University of Illinois Urbana–Champaign. Roughly 1 ng of template DNA from each sample was amplified using the V4 primer pair—V4_515F (5’-GTGYCAGCMGCCGCGGTAA-3’) and V4_806R (5’-GGACTACNVGGGTWTCTAAT-3’) (Apprill et al., 2015; Parada et al., 2016)—following the Fluidigm standard protocol (Standard BioTools, CA). Barcoded amplicons generated for each sample were quantified with a Qubit fluorometer (ThermoFisher Scientific, CA, USA), and fragment sizes were assessed using a Fragment Analyzer (Agilent, CA). Amplicons were then pooled at equimolar concentrations, separated on a 2% agarose Ex-gel (ThermoFisher Scientific, CA, USA) to eliminate primer dimers, and purified from the gel using a Qiagen gel extraction kit.

The cleaned, size-selected pool was re-quantified and re-evaluated on the Fragment Analyzer to verify fragment size distribution. Afterward, the pooled library was diluted to 5 nM and its concentration further refined via qPCR using a CFX Connect Real-Time system (Bio-Rad, Hercules, CA, USA) to optimize cluster density on the sequencing flow cell. The pooled library was denatured, supplemented with 20% non-indexed PhiX v3 control (Illumina, CA, USA), and then loaded onto a MiSeq v2 flow cell (500-cycle kit) at 8 pM to enable cluster generation and sequencing. Paired-end sequencing was performed, yielding 250-nt reads from both directions. Raw sequencing output was processed with bcl2fastq v2.20 (Illumina, CA, USA) to generate demultiplexed, compressed FASTQ files.

### 2.3 Illumina reads processing

Illumina forward and reverse sequences were processed with the DADA2 pipeline (Callahan et al., 2016) using R version 4.4.2 in RStudio (R Core Team, 2021). Forward and reverse paired-end reads were quality-filtered, trimmed, and truncated with the *filterAndTrim* function, applying the parameters maxN = 0, maxEE = c(2, 5), and truncQ = 1. Error profiles were then learned with *learnErrors*, and amplicon sequence variants (ASVs) were inferred using the core *dada* algorithm. Chimeric sequences were detected and removed via *removeBimeraDenovo* with the “consensus” method. Taxonomy was assigned using the SILVA reference database (silva_nr99_v138.2_toSpecies_trainset).

To address mismatches within primer-binding regions that can introduce variability in read lengths, DADA2-generated amplicon sequence variants (ASVs) were aligned using the MUSCLE algorithm in SeaView (Galtier et al., 1996; Gouy et al., 2010; Mirarab et al., 2015). The resulting alignment was imported into MEGA6 (Tamura et al., 2013) for visual quality control of primer placement relative to the expected amplicon boundaries. Based on the reference alignment, primer-binding regions were consistently trimmed across all ASVs at fixed positional boundaries to ensure uniform recovery of the target locus. Following trimming, identical ASVs resulting solely from differences in sequence length at the primer boundaries were collapsed using DECIPHER (Wright, 2016) at 100% sequence similarity to remove redundancy introduced by alignment-based trimming. ASVs detected in negative controls were removed prior to downstream analyses to account for potential laboratory or reagent contamination. Finally, ASV abundances were normalized using Total Sum Normalization (proportional abundance per sample), calculated as the abundance of each ASV divided by the total number of reads per sample (McKnight et al., 2019).

Phylogenetic analysis was performed in SeaView using the neighbor-joining approach (Saitou and Nei, 1987). The resulting tree was visually inspected to confirm that sequences grouped with appropriate taxa and to identify unusually long branches that could suggest non-bacterial ASVs. Long branch ASVs were compared to the GenBank database, and those matching bacterial lineages were retained, whereas those of non-bacterial origin were removed. Finally, taxonomic nomenclature followed the SILVA database convention.

### 2.4 Community analyses

All analyses were performed in version 4.4.2 in RStudio (R Core Team, 2021). The ASVs detected in negative controls were removed from all samples prior to downstream analyses to minimize potential laboratory contamination. Rarefaction curves generated with the vegan package (Dixon, 2003; Oksanen et al., 2016) were used to assess sampling depth. Samples were not rarefied, and downstream analyses were conducted on normalized relative abundance data. ASV abundances were normalized using total sum scaling (relative abundance per sample). Heat maps were generated from relative abundance data using the pheatmap package (Kolde, 2019).

Alpha diversity metrics were calculated to assess within-sample diversity. Observed ASV richness and Faith’s phylogenetic diversity (PD) were computed using the picante (Kembel et al., 2010), pez (Pearse et al., 2015), and vegan (Dixon, 2003) packages. Standardized effect sizes of phylogenetic diversity (SES.PD) were calculated by comparing observed PD values to null distributions generated through randomization procedures (999 permutations). Differences in alpha diversity metrics (observed ASV richness and SES.PD) among host age (juvenile vs. adult), host sex (male vs. female), and human population density (urban vs. rural) were assessed using Wilcoxon rank-sum tests. Values are reported as mean ± standard deviation. Statistical significance was defined as p < 0.05.

The influence of host age (juvenile vs. adult), host sex (male vs. female), and human population density (urban vs. rural) on bacterial community composition was assessed using multiple complementary approaches (Ramette, 2007). Beta-diversity based on relative abundance data was calculated using Bray–Curtis dissimilarity matrices. Differences in community composition were tested using permutational multivariate analysis of variance (PERMANOVA; *adonis* function) and analysis of similarities (ANOSIM) within the vegan package (Dixon, 2003), each performed with 1,000 permutations. Each factor (host age, host sex, and human population density) was tested independently using separate PERMANOVA models. The *envfit* function was used to assess the association between explanatory variables and ordination structure. ANOSIM R-values (−1 to 1) were interpreted as indicators of between-group versus within-group dissimilarity. Homogeneity of multivariate dispersion was assessed using the *betadisper* function with bias adjustment (Gerhard et al., 2022) to confirm that differences detected by PERMANOVA were not driven by unequal dispersion among groups.

The phylogenetic tree used for all phylogenetic analyses was reconstructed in PASTA v1.6.1 (Mirarab et al., 2015) using the following settings: Aligner = MAFFT, Merger = MUSCLE, Tree Estimator = FastTree, Model = GTR+G20, and Decomposition = centroid. The resulting phylogenetic tree was used for all phylogenetic diversity and UniFrac-based analyses. Phylogenetic beta-diversity was evaluated using generalized UniFrac distances (α = 0.5) implemented in the GUniFrac package (Chen et al., 2012), and statistical significance was assessed using PERMANOVA with 1,000 permutations. This generalized UniFrac approach (α = 0.5) incorporates both phylogenetic relationships and relative abundance information and has been shown to be more robust than both unweighted and weighted UniFrac distance measures (Chen et al., 2012). Bray–Curtis dissimilarities were used for taxonomic beta-diversity analyses, whereas generalized UniFrac distances were used for phylogenetic beta-diversity analyses. Phylogenetic beta-diversity analyses were conducted using ASV-level phylogenetic relationships, with results summarized at the order level.

Community composition was visualized using non-metric multidimensional scaling (NMDS) based on Bray–Curtis dissimilarities and transformation-based principal component analysis (tb-PCA). The tb-PCA was conducted on Hellinger-transformed ASV data following Legendre and Gallagher (2001). Presence–absence data were generated by converting ASV abundances to binary values (0 = absent, 1 = present), and these binary-transformed data were subsequently Hellinger-transformed prior to tb-PCA. This transformation is appropriate for community composition analyses and reduces the influence of highly abundant taxa.

## 3 Results

### 3.1 DNA extraction, Illumina sequencing run metrics, and alpha-diversity metrics

DNA extraction yielded an average concentration of 3.5 ng/μL (range: 0.01–55 ng/μL). A total of 106 samples and 2 negative controls were processed in a single Illumina sequencing run, generating 15,008,466 paired-end reads and resulting in 15,031 ASVs. Taxonomic assignment indicated that 11,779 ASVs (78.4%) were classified within the Kingdom Bacteria. Alpha diversity metrics did not differ significantly across host age or sex (**Table 1**). Observed ASV richness was significantly higher in rural compared to urban samples, whereas standardized phylogenetic diversity (SES.PD) did not differ between environments (**Table 1**).

**Table 1:** Alpha diversity metrics (observed ASV richness and standardized effect sizes of phylogenetic diversity, SES.PD) across host traits and environmental categories.

Comparison	Metric	Group 11	Group 21	Statistic	P-value
Age (Adult vs Juvenile)	Richness	212.13 ± 99.06	187.02 ± 81.14	1563.5	0.22
	SES.PD	−5.82 ± 1.96	−5.45 ± 1.94	1224	0.34
Sex (Female vs Male)	Richness	193.13 ± 85.03	202.40 ± 94.72	1355	0.76
	SES.PD	−5.78 ± 1.86	−5.43 ± 2.04	1234	0.28
Environment (Rural vs Urban)	Richness	215.24 ± 94.98	167.51 ± 71.01	1710	*0.008
	SES.PD	−5.50 ± 2.15	−5.79 ± 1.56	1425	0.44

^1^ Values are presented as mean ± standard deviation. Statistical significance was assessed using Wilcoxon rank-sum tests. * Indicates statistically significant p-value.

### 3.2 Taxonomic composition and distribution

The goose cloacal microbiota contained bacteria from 29 phyla, 56 classes, and at least 131 orders. The four prominent phyla were Bacillota (46.7% [5506/11779]), Actinomycetota (16.4% [1930/11779]), Pseudomonadota (15.3% [1805/11779]), and Bacteroidota (9.7% [1145/11779]). Within the phylum Bacillota, the class Clostridia was the most prominent (66.9% [3682/5506]), followed by the class Bacilli (28.6% [1572/5506]). The class Actinobacteria accounted for 71% of the ASVs within the phylum Actinomycetota, and the classes Alphaproteobacteria and Gammaproteobacteria accounted for 44.8% and 53.6% of the phylum Pseudomonadota, respectively. The class Bacteroidia accounted for 100% of the phylum Bacteroidota.

**Figure 2:**
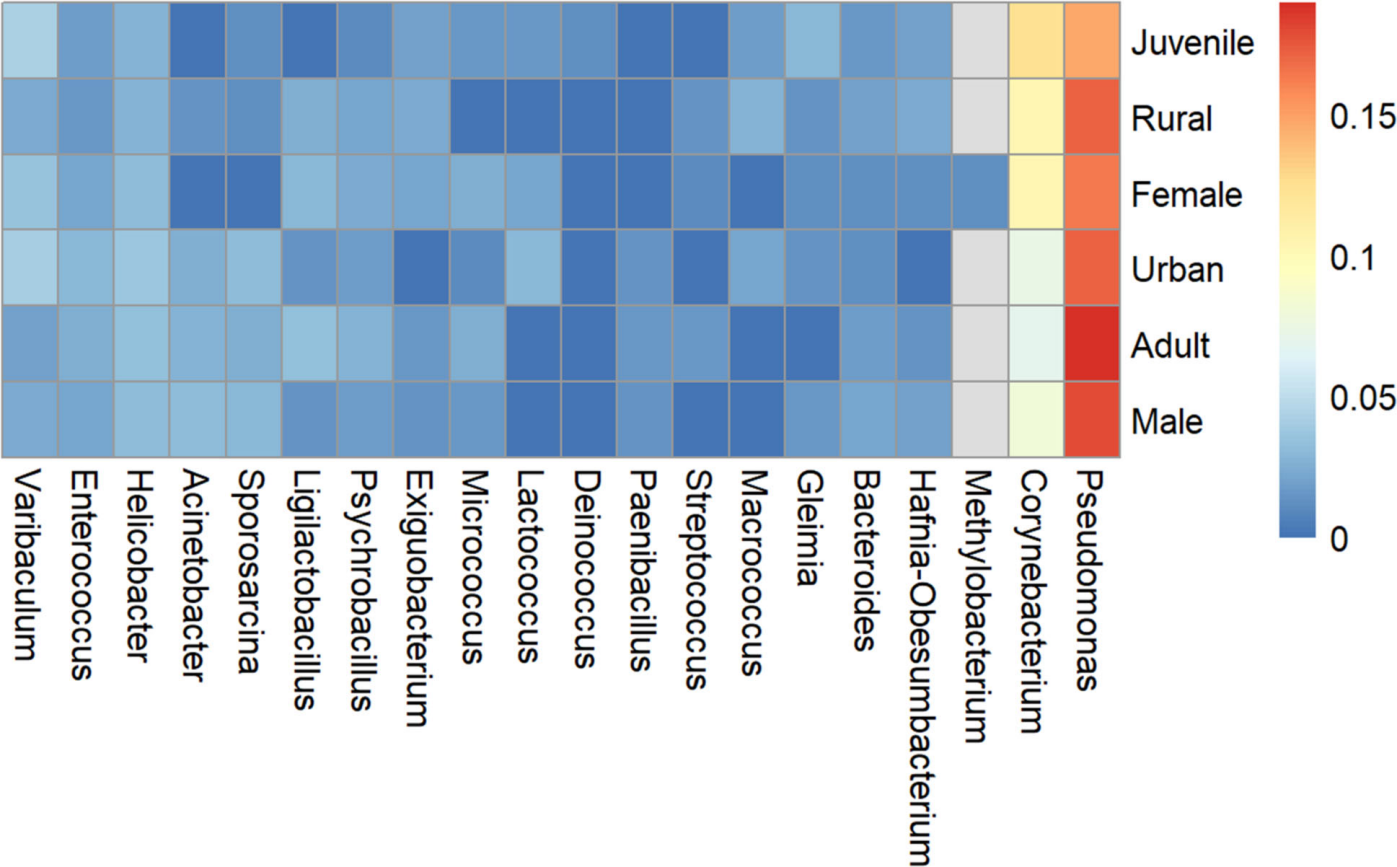
Heat map of the top fifteen most abundant genera per category found in Canada Goose (*Branta canadensis*) cloacal samples from Illinois based on 16S rRNA gene V4 region.

The most common genera were *Pseudomonas* and *Corynebacterium* (**Figure 2**). Across all individual geese, *Corynebacterium* was the most prevalent genus (89.6%, 95/106 individuals), followed by *Pseudomonas* (86.8%, 92/106). Additional prevalent genera included *Bacteroides* (84.9%, 90/106), *Varibaculum* (79.2%, 84/106), *Methylobacterium* (78.3%, 83/106), *Deinococcus* (77.4%, 82/106), and *Ligilactobacillus* (75.5%, 80/106). The cloacal samples contained environmental, plant-associated (diet), and common gut-associated genera. Acidibacter, Acidisoma, and Acidiferrimicrobium were detected, as well as several genera within the family Rhizobiaceae (*Agrobacterium, Allorhizobium, Ensifer, Neorhizobium, Pararhizobium, Rhizobium*) and other nitrogen-fixing genera (*Bradyrhizobium* and *Azorhizobium*). Common gut-associated genera included *Clostridium*, *Escherichia*, *Eubacterium*, and *Ruminococcus*.

The potential human-associated taxon *Clostridioides difficile* was detected in one urban sample (0.12888% relative abundance) and one rural sample (0.001419% relative abundance). *Gleimia* and *Sporosarcina* exhibited higher relative abundance in urban samples, with *Gleimia* higher in juveniles and *Sporosarcina* higher in adult males (**Figure 2**).

### 3.3 Beta-diversity metrics

**Figure 3:**
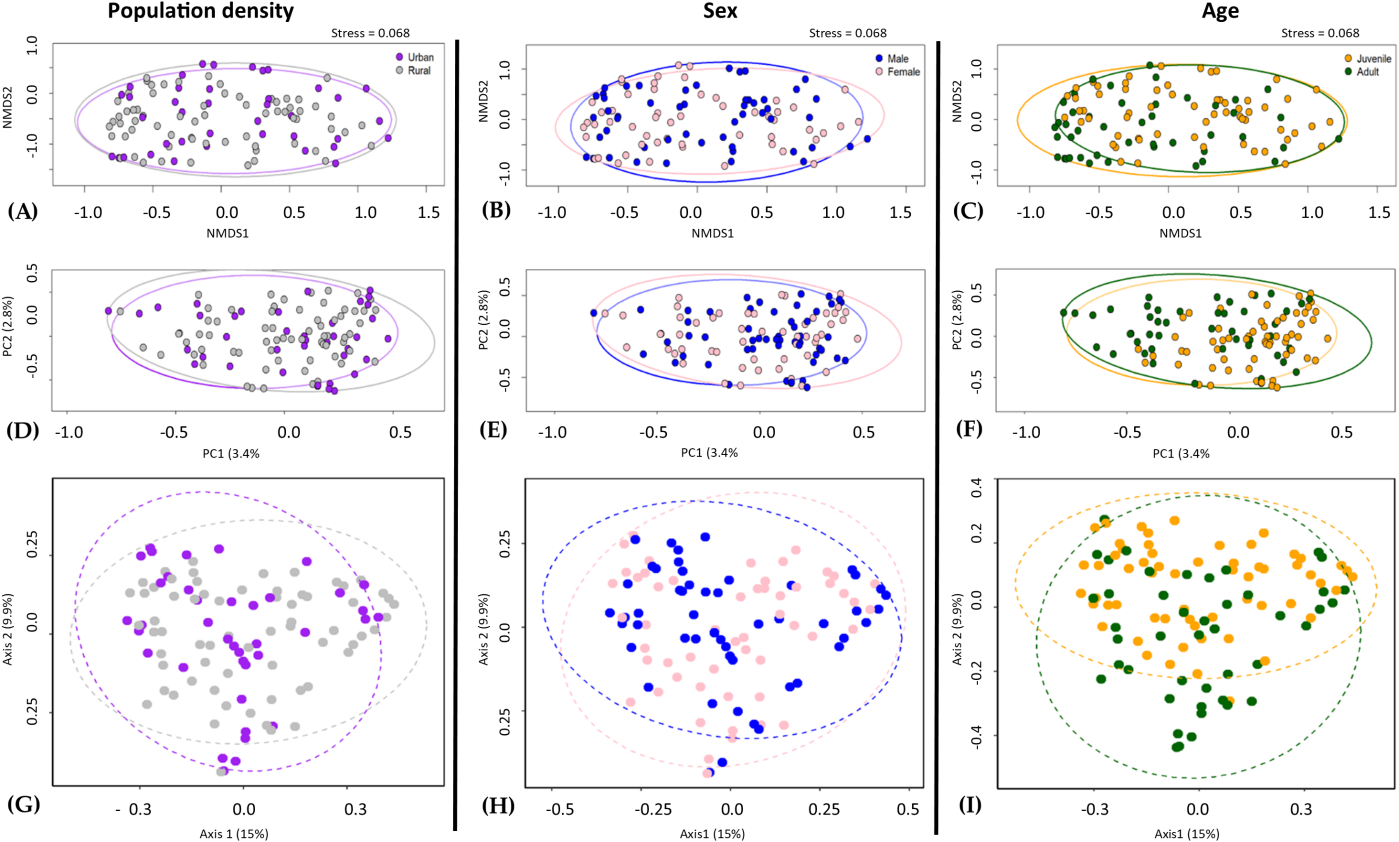
Visualization of bacterial beta-diversity from cloacal samples of Canada goose (*Branta canadensis*) in Illinois based on the V4 region of the 16S rRNA gene. **(A–C)** Non-metric multidimensional scaling (NMDS) ordinations based on Bray–Curtis dissimilarities of ASV relative abundance data; **(D–F)** transformation-based principal component analysis (tb-PCA) of Hellinger-transformed ASV data; **(G–I)** principal coordinates analysis (PCoA) based on generalized UniFrac (α = 0.5) distances at the order level. Ellipses represent the convex hulls, indicating the overall spread and extent of variation within each group.

**Figure 4:**
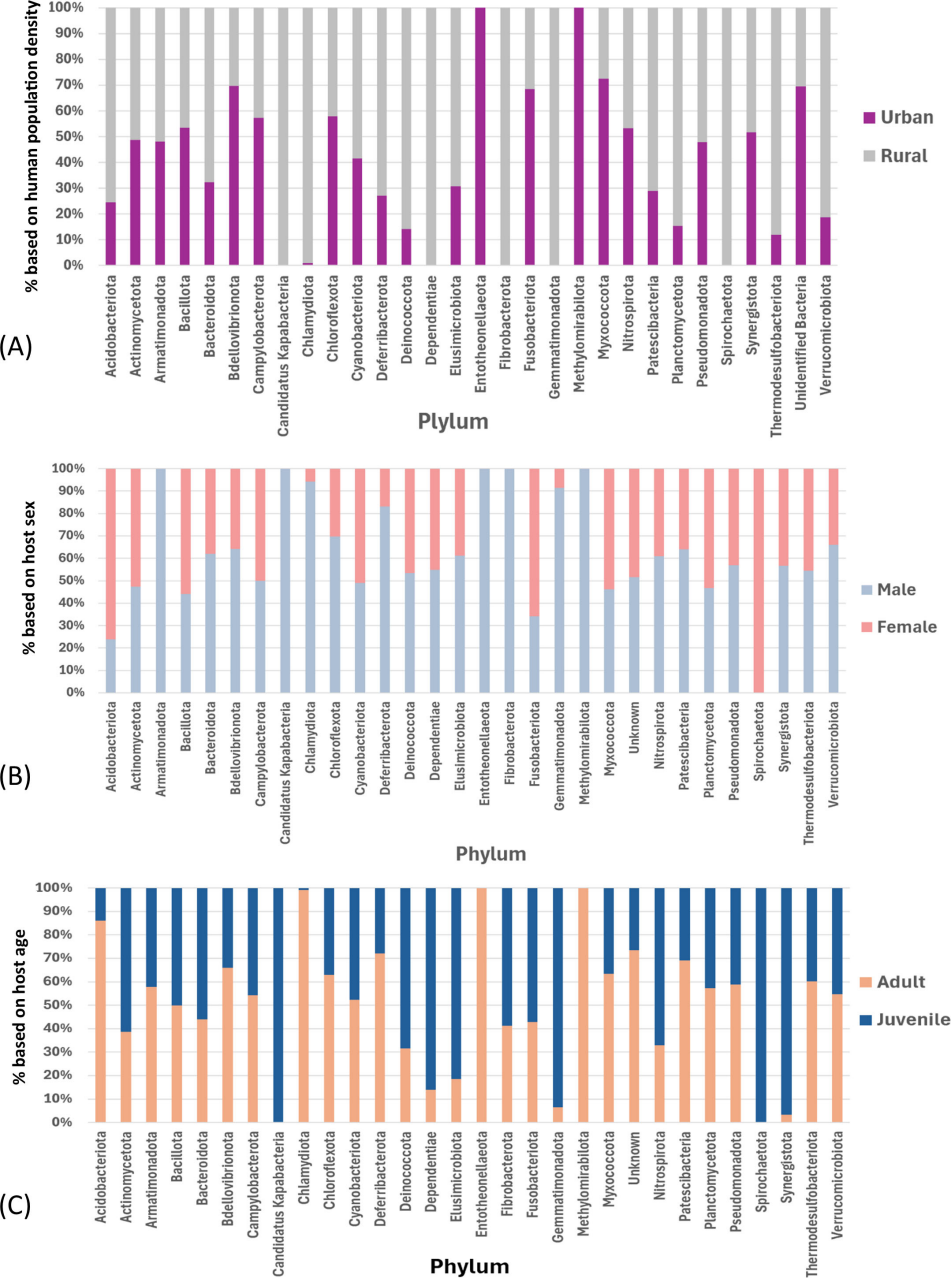
Phylum level distribution of the bacterial community from cloacal samples from Canada goose (*Branta canadensis*) in Illinois. The Y axis represents the % relative abundance per phylum **(A)** Distribution by human population density (by location, urban or rural); **(B)** Distribution by host sex; **(C)** Distribution by host age.

Analyses of both relative abundance and presence–absence datasets indicated that host age and human population density (rural vs. urban) were significantly associated with differences in microbial community composition. Host age was significant across all statistical approaches (envfit, PERMANOVA, and ANOSIM), whereas human population density was significant in two of three analyses (ANOSIM and PERMANOVA) (**Table 2**; **Figure 3**). Phylogenetic beta-diversity differed significantly with host age (PERMANOVA: df = 1, R² = 0.026, p = 0.001) and human population density (df = 1, R² = 0.015, p = 0.037), but not with host sex (df = 1, R² = 0.007, p = 0.80) (**Figure 3**). At the phylum level, Entotheonellaeota and Methylomirabilota were detected only in a single adult urban male, whereas Chlamydiota was repeatedly observed, predominantly in adult rural males (**Figure 4**). In contrast, Spirochaetota was detected only in a single juvenile rural female (**Figure 4**).

**Table 2:** Factors that affect the overall bacterial beta-diversity (variation in species composition) of the Canada goose (*Branta canadensis*) cloacal samples from Illinois.

Factor/data set	Analysis	Relative abundance			Presence/absence		
		R2 value	R value	P value	R value	R value	P value
Population density	envfit	0.023	NA	0.071	0.003	NA	0.659
	adonis	0.016	NA	*0.013	0.013	NA	*0.007
	anosim1	NA	0.07	*0.015	NA	0.067	*0.028
Host age	envfit	0.045	NA	*0.003	0.124	NA	*0.001
	adonis	0.025	NA	*0.001	0.019	NA	*0.001
	anosim	NA	0.053	*0.012	NA	0.064	*0.007
Host sex	envfit	0.016	NA	0.184	0.004	NA	0.646
	adonis	0.007	NA	0.853	0.009	NA	0.682
	anosim	NA	-0.009	0.758	NA	0.001	0.418

^1^ The anosim generated R-values ranging from −1 to 1, with values near 1 indicating greater dissimilarity between groups than within groups. Significant *P*-values in bold. NA, non-applicable.

## 4 Discussion

This study provides one of the first comprehensive characterizations of the cloacal bacterial microbiota of resident Canada goose populations in Illinois, USA, and explores how host traits and human population density are associated with variation in microbiota composition and ASV richness. Using high-throughput 16S rRNA gene sequencing, our results indicate that Illinois geese harbor diverse bacterial communities, including taxa (at the genus level) commonly associated with vertebrate guts, environmental sources, and diet. Collectively, our findings indicate that host age and human population density were associated with differences in microbial community composition, whereas alpha diversity metrics were largely consistent across host traits, except for higher ASV richness in rural compared to urban samples.

While observed ASV richness and standardized phylogenetic diversity (SES.PD) did not differ significantly between age classes, both groups exhibited consistently negative SES.PD values, indicating phylogenetic clustering relative to null expectations. In contrast, beta-diversity analyses revealed significant shifts in community composition with host age and, to a lesser extent, with human population density. The lack of significant differences in alpha diversity alongside significant differences in beta-diversity indicates that community composition varies across groups despite relatively stable overall diversity. Notably, rural samples exhibited higher ASV richness than urban samples; however, this increase did not correspond to differences in phylogenetic structure, suggesting that additional taxa are drawn from similar evolutionary lineages. Although these associations were statistically significant, the relatively low R² values indicate that these factors explain only a small proportion of the total variation in community composition.

However, these interpretations should be considered with caution due to the potential effects of pseudoreplication and the resulting low R² values. If samples were not fully independent, for example due to repeated sampling within the same locations or shared environmental conditions, this could inflate the apparent strength of observed patterns. As a result, the detected associations between microbiota structure and factors such as age or human population density may, in part, reflect site-specific or population-level effects rather than broadly generalizable processes. Consequently, our findings are best interpreted as being constrained to the sampled populations in Illinois and may not fully extend to other Canada goose populations or geographic regions without further replication across independent sites and populations.

### 4.1 Microbiota composition and comparison with other avian systems

The dominant phyla observed in our samples included Bacillota, Actinomycetota, Pseudomonadota, and Bacteroidota. These phylum-level patterns mirror those reported across studies of wild and captive birds, suggesting a conserved backbone of avian gut microbiota despite ecological differences among host species (Waite and Taylor, 2015; Wang et al., 2019). Such broad similarities have been documented in reviews and comparative studies of avian gut bacterial communities (Grond et al., 2018; Sun et al., 2022). The presence of the class Clostridia and members of Bacteroidia suggests functional roles in anaerobic fermentation and polysaccharide degradation, consistent with their roles in other vertebrate guts (Bentley and Meganathan, 1982; Thomas et al., 2011). The frequent detection of genera such as *Pseudomonas*, *Corynebacterium*, *Micrococcus*, and *Acinetobacter* is also consistent with findings from other waterfowl (Grond et al., 2018) and urban-adapted bird studies, where environmental exposure to human activities influences avian gut bacterial community composition (Phillips et al., 2018).

The genus *Sporosarcina* has been suggested to play a potentially important role in gut microbiota, as some species may function as probiotics by promoting anti-inflammatory responses, inhibiting pathogens, and supporting nutrient metabolism (Kehri et al., 2023). *Sporosarcina* is commonly reported in Canada goose cloacal and fecal samples (Kehri et al., 2023) and has also been identified across a range of hosts, including poultry, other waterfowl, and wild species such as raptors (Zhou et al., 2020). Consistent with these findings, *Sporosarcina* was frequently detected in Illinois Canada goose in this study, appearing largely independent of sampling area or host age. However, *Sporosarcina* was not commonly observed in female geese within our sampling locations. Differences such as these could potentially be influenced by intrinsic factors (e.g., hormonal variation or reproductive physiology) as well as extrinsic factors (e.g., behavioral or social differences), which have been shown to shape microbiota composition in other systems (Grond et al., 2018). That said, these explanations remain speculative, and additional targeted research would be necessary to determine the mechanisms underlying the observed patterns.

Studies of Canada goose fecal samples have suggested that gut microbiota composition may vary more strongly with geographic distance than with host-associated factors (Gil et al., 2025). Our results using Canada goose cloacal samples appear to be broadly consistent with this pattern, highlighting the idea that large geographic distances may play a more important role in shaping cloacal and fecal microbiota among Anseriformes, such as Canada goose, than host traits like age or sex. For example, only two of the 20 most common genera across all categories identified in Illinois geese cloacal samples (**Figure 2**) overlapped with those reported at a broader, nationwide scale (Gil et al., 2025). These genera (*Helicobacter* and *Bacteroides*) are typically considered commensal and were detected consistently across Illinois samples, regardless of location, sex, or age (**Figure 2**). However, while *Helicobacter* was reported across multiple regions nationwide, *Bacteroides* appeared to be more geographically restricted, occurring only in geese sampled along the Atlantic flyway (e.g., Rhode Island, Connecticut, New York, and New Jersey; Gil et al., 2025). Taken together, these patterns further highlight the potential importance of geographic distance and regional environmental variation when comparing microbiota across avian populations, although differences in sampling design and methodology among studies should also be considered when interpreting these comparisons.

### 4.2 Host age-related patterns

Our results indicate that alpha diversity metrics did not differ significantly between juvenile and adult Canada goose, although differences in microbial community composition were observed between age classes. This pattern is broadly consistent with previous research indicating that microbial community structure may shift across life stages even when overall diversity remains stable. For example, González-Braojos et al. (2012) and Waite and Taylor (2015) found that the gut microbiota of young altricial birds differ from those of adults, while in precocial species (those born relatively independent) local environmental exposure is thought to play a substantial role in microbiota acquisition, with more limited direct parental influence (Grond et al., 2017).

Because Canada goose is precocial, both environmental exposure and parental influence may contribute to microbiota assembly, which could partially explain the relatively low R² values observed when comparing diversity between juvenile and adult individuals. This suggests that age alone may not be a strong predictor of overall diversity metrics in this population, or that additional unmeasured factors may be influencing these patterns. The observed differences in microbiota composition between age classes could have implications for disease susceptibility and transmission, although such effects cannot be determined from the present data.

### 4.3 Urbanization, habitat, and microbial diversity

Geographic context, particularly urban versus rural habitat, was associated with differences in microbial community composition, as supported by beta-diversity analyses. Human population density was identified as a significant factor in PERMANOVA and ANOSIM, although the proportion of variance explained was relatively low, indicating that urbanization contributes to, but does not solely drive, community structure. In contrast, host age showed a stronger and more consistent association across all statistical approaches. Rural geese also exhibited higher ASV richness than urban individuals, although this pattern was not accompanied by differences in alpha-phylogenetic diversity. This suggests that while taxonomic richness may decline in more urbanized environments, broader phylogenetic structure remains relatively conserved. Similar patterns have been reported in other avian systems, where urbanization is associated with shifts in microbial composition and reductions in richness, potentially linked to differences in habitat heterogeneity, diet, and exposure to anthropogenic factors (Phillips et al., 2018; Dillard et al., 2022). The detection of members of the Rhizobiaceae and other nitrogen-fixing genera across samples is consistent with environmental acquisition during foraging and further supports the influence of habitat and diet on microbial assemblages (Graham and Vance, 2003; Poole et al., 2018). Additionally, the occurrence of low-frequency, environment-associated phyla in specific host and habitat contexts highlights the potential for localized or stochastic inputs to contribute to observed community differences. Overall, these findings indicate that urbanization is one of several interacting factors shaping microbial community composition in Canada goose.

### 4.4 Public-health implications

Low-abundance sequences from genera that may include human opportunistic pathogens (e.g., *Acinetobacter, Bacteroides, Streptococcus, Hafnia*, and *Clostridioides*) were detected across samples. The rare detection of *Clostridioides difficile* in two samples is notable; however, amplicon-based sequencing alone cannot determine strain identity, virulence potential, or organism viability. As such, these detections most likely reflect low-level environmental exposure or transient passage through the gastrointestinal tract rather than established colonization or infection. In our samples, *Escherichia coli* was detected at low relative abundance in four samples (3.8%, 4/106). This pattern is consistent with Gil et al. (2025), who reported *E. coli* in only 9.9% (16/161) of their samples.

More broadly, the repeated detection of genera such as *Acinetobacter, Streptococcus, Bacteroides*, and *Clostridioides*, even at low relative abundance, identifies these taxa as candidates for targeted follow-up studies. Their consistent presence across individuals suggests that they may serve as useful indicators of environmental microbial exposure in human-influenced landscapes. Future work integrating culture-based approaches, quantitative PCR (qPCR), and higher-resolution genomic methods will be necessary to determine strain-level identity, assess potential pathogenicity, and evaluate whether these taxa represent transient environmental inputs or stable members of the cloacal microbiota.

Interpretation of low-abundance taxa should also be considered in the context of contamination control procedures, as filtering approaches designed to remove laboratory or reagent-derived contaminants may also exclude taxa present near the detection threshold. Taken together, these findings highlight a subset of bacterial genera that are consistently detectable in Canada goose cloacal samples and may provide a focused framework for future studies examining microbial transmission, environmental reservoirs, and potential public health relevance.

### 4.5 Limitations and recommendations for future work

This study relied on cloacal swabs and 16S rRNA gene V4 region amplicon sequencing, which limit functional inference and the ability to distinguish among host-associated, environmental, and diet-derived taxa. Previous studies suggest that fecal sampling may more accurately represent the distal colon or lower gastrointestinal microbiota (Drovetski et al., 2018). Accordingly, future work should incorporate fecal sampling and longitudinal sampling across life stages to evaluate temporal stability and microbiota maturation.

The removal of ASVs detected in negative controls represents an important step to minimize laboratory and reagent contamination; however, this approach may also exclude low-abundance taxa that are biologically present in samples, including those associated with human or environmental sources. As a result, some human-associated microbes may be underrepresented, particularly near the detection threshold. In this study, only a small number of genera associated with negative controls were removed (*Microcella, Succinivibria*, and *Prevotella*), all of which include human-associated species, suggesting that this filtering step may have had a limited but measurable impact on the detection of low-abundance taxa.

Integrating targeted culture-based methods or quantitative PCR (qPCR) for putative pathogenic taxa would further enable strain-level identification and characterization of virulence determinants and antimicrobial resistance genes. Expanding geographic sampling to reduce the potential influence of pseudoreplication and implementing recapture efforts would provide valuable insights into bacterial community stability and facilitate the identification of microbial markers relevant to long-term monitoring. In parallel, incorporating environmental sampling would strengthen our understanding of the potential sources from which geese acquire their microbiota. Finally, although the V4 region provides robust genus-level resolution suitable for community-level comparisons, full-length 16S rRNA gene sequencing using long-read approaches will enhance discrimination among closely related taxa and allow for more definitive species-level identification.

## 5 Summary

In summary, this study provides an initial characterization of the cloacal microbiota of resident Canada goose in Illinois and identifies associations between bacterial community composition and host age, as well as between bacterial community composition and human population density across urban–rural gradients. While alpha diversity metrics were largely consistent across host traits, except for higher ASV richness in rural compared to urban samples, beta-diversity analyses revealed significant differences in community composition, particularly with host age. These findings indicate that microbial community composition varies across ecological and host-associated gradients despite relatively stable overall diversity. Although consistent patterns were detected, the relatively modest explanatory power of the models suggests that additional unmeasured environmental and spatial factors likely contribute to microbiota variation.

Importantly, the repeated detection of specific bacterial genera, including *Acinetobacter, Streptococcus, Bacteroides*, and *Clostridioides*, highlights a subset of taxa that may serve as useful targets for future studies investigating environmental microbial exposure and potential public-health relevance. Overall, these findings support the idea that Canada goose microbiota is more strongly associated with local ecological context than with host intrinsic factors alone, particularly with respect of community composition; however, the scope of inference remains limited to the sampled populations. Further work incorporating broader geographic replication, longitudinal sampling, and environmental comparisons will be needed to more fully resolve the drivers and stability of these microbial communities.

## Data Availability

The datasets presented in this study can be found in online repositories. The names of the repository/repositories and accession number(s) can be found below: https://www.ncbi.nlm.nih.gov/genbank/, PRJNA1426908.
